# Epidemiology and outcomes of sepsis among hospitalizations with systemic lupus erythematosus admitted to the ICU: a population-based cohort study

**DOI:** 10.1186/s40560-019-0424-y

**Published:** 2020-01-06

**Authors:** Lavi Oud

**Affiliations:** Division of Pulmonary and Critical Care Medicine, Department of Internal Medicine, Texas Tech University Health Sciences Center at the Permian Basin, Odessa, TX 79763 USA

**Keywords:** Systemic lupus erythematosus, Sepsis, Critical illness, Intensive care unit, Mortality

## Abstract

**Background:**

Sepsis is the most common cause of premature death among patients with systemic lupus erythematosus (SLE) aged ≤ 50 years in the United States, and infection is the most common cause of admission to the ICU among SLE patients. However, there are no population-level data on the patterns of the demand for critical care services among hospitalized septic patients with SLE or the outcomes of those admitted to the ICU.

**Methods:**

We performed a retrospective cohort study, using the Texas Inpatient Public Use Data File, to identify SLE hospitalizations aged ≥ 18 years and the subgroups with sepsis and ICU admission during 2009–2014. The patterns of ICU admission among septic hospitalizations were examined. Logistic regression modeling was used to identify predictors of short-term mortality (defined as hospital death or discharge to hospice) among ICU admissions with sepsis and to estimate the risk-adjusted short-term mortality among ICU admissions with and without sepsis.

**Results:**

Among 94,338 SLE hospitalizations, 17,037 (18.1%) had sepsis and 9409 (55.2%) of the latter were admitted to the ICU. Sepsis accounted for 51.5% of the growth in volume of ICU admissions among SLE hospitalizations during the study period. Among ICU admissions with sepsis, 25.3% were aged ≥ 65 years, 88.6% were female, and 64.4% were non-white minorities. The odds of short-term mortality among septic ICU admissions were increased among those lacking health insurance (adjusted odds ratio 1.40 [95% confidence interval 1.07–1.84]), while being unaffected by gender and race/ethnicity, and remaining unchanged over the study period. On adjusted analyses among ICU admissions, the short-term mortality among those with and without sepsis was 13% (95% CI 12.6–13.3) and 2.7% (95% CI 2.6–2.8), respectively. Sepsis was associated with 63.6% of all short-term mortality events.

**Conclusions:**

Sepsis is a major, incremental driver of the demand for critical care services among SLE hospitalizations. Despite its relatively low mortality, sepsis was associated with most of the short-term deaths among ICU patients with SLE.

## Background

Systemic lupus erythematosus (SLE) is a multisystem autoimmune disease of an unknown etiology [1]. The long-term survival of SLE has markedly improved over the past decades [2], but the risk of premature death has plateaued since 1999 [3] and considerable morbidity continues to afflict affected patients [4]. Patients with SLE are commonly hospitalized [5] and SLE has become over the past two decades the most common autoimmune disease in the ICU [6]. The substantial immune dysfunction among patients with SLE increases their risk of serious infectious complications [7]. Infections in SLE patients can be in turn difficult to distinguish from disease flare-ups [8], while immunosuppressive therapy can change the initial manifestations of infection. Together, these latter factors may lead to delayed diagnosis of infection and subsequent sepsis, thus increasing the risk of lethal outcomes. Indeed, the standardized mortality rate due to infection was found to be five times higher in SLE patients than in the general population [9].

Infections are reported as the most common cause of ICU admission of patients with SLE [6], with frequency ranging from 14% [10] to 82% [11]. However, it is the development of sepsis among infected patients that is a key driver of morbidity, mortality [12, 13] and increased use of healthcare resources [14], including critical care [15] in the general population and likely in SLE. Indeed, in a recent study of SLE decedents aged ≤ 50 years in the United States (US), sepsis has been the most common cause of premature death [16].

Thus, contemporary data on the demand for critical care services among septic SLE patients and the characteristics and outcomes of those requiring ICU care can inform decisions on resource allocation, preventive and interventional efforts to improve patient outcomes, and provide benchmarks for performance improvement initiatives.

Numerous studies have described infections among ICU-managed SLE patients [11, 17–23]. However, sepsis affects only about half of ICU patients with an infection in the general population [15] and inferences about sepsis in studies of ICU-managed SLE patients with infections are limited in part because investigators have often conflated infection and sepsis. Thus, sepsis was either not mentioned [19], not defined [17], used interchangeably with infection [22], or used as a category of site-specific infections [20]. The generalizability of prior studies is further affected by use of single-centered [11, 17–19, 22, 23], small cohort data [11, 17–19, 21, 22], with most of the data being over a decade old [11, 17–20, 23]. Finally, sepsis was generally not the primary focus of most studies of ICU-managed SLE patients to date [11, 17–20, 22, 23]. Only two population-based studies have focused, to our knowledge, on sepsis among ICU patients with SLE, with one reporting only on SLE hospitalizations with septic shock in France, of which 95% were admitted to the ICU [24], while another described a small registry-based cohort in Israel [21]. Finally, the impact of sepsis on hospital resource utilization among critically ill SLE patients has not been systematically examined.

In order to address the noted knowledge gaps, we performed a population-based study of contemporary ICU-managed of adults with SLE to (a) examine the patterns of critical care services utilization among septic patients, (b) quantify the impact of sepsis on hospital resource utilization and short-term mortality among critically ill patients with SLE, and (c) determine the factors associated with short-term mortality among those with sepsis in the latter group.

## Materials and methods

This was a retrospective, population-based cohort study. Because we used a publicly available, de-identified dataset, the study was determined to be exempt from formal review by the Texas Tech Health Sciences Center’s Institutional Review Board. The reporting of the study finding followed the STROBE guidelines on reporting observational studies in epidemiology [25].

### Data sources and study population

We used the Texas Inpatient Public Use Data File (TIPUDF) to identify the target population. In brief, the TIPUDF is an administrative dataset maintained by the Texas Department of State Health Services [26] and includes inpatient discharge data from state-licensed, non-federal hospitals and captures approximately 97% of all hospital discharges in the state.

We identified hospitalizations of patients aged ≥ 18 years between the years 2009 and 2014 with a diagnosis of SLE, based on the presence of International Classification of Diseases, Ninth Revision, Clinical Modification (ICD-9-CM) code 710.0 in the principal or secondary diagnosis fields. This code was validated in administrative data, with sensitivity of 79.3%, specificity of 90.2%, and with a positive predictive value of 95.9% [27], and was used in prior studies of SLE [28, 29]. Hospitalizations with sepsis were identified by the presence of either (a) a combination of ICD-9-CM codes for infection and one or more organ dysfunctions, as described by Angus et al. [30], or (b) “explicit” codes for severe sepsis (995.92) or septic shock (785.52). This approach was found to combine maximal sensitivity and specificity for identification of sepsis in administrative data in a chart-based validation study, as compared with other ICD-based algorithms [31], has comparable sensitivity to sepsis identified in electronic medical records [32], and has been used in contemporary studies of sepsis in administrative data to align ICD-based algorithms with the framework of Sepsis-3 [32, 33]. The abovementioned “explicit” code for septic shock has been used to describe septic shock in the present study [34, 35]. ICU admissions were identified based on unit-specific revenue codes for an intensive care unit or a coronary care unit. The TIPUDF dataset does not include information on the indications or specific diagnoses for admission to the ICU.

### Outcomes

The primary outcome was the short-term mortality among SLE hospitalizations with sepsis who were admitted to the ICU. Short-term mortality was defined as the combination of in-hospital mortality or discharge to hospice. We chose this definition of short-term mortality instead of hospital mortality because discharge to hospice is increasingly common among septic patients and there is a potential for overestimation of short-term survival of septic populations when examined over time, if discharges to hospice are not considered [32].

The secondary outcomes included (a) rates of ICU admission among all SLE hospitalizations and those with sepsis, (b) rates of sepsis among ICU admissions with SLE, and (c) hospital length of stay and hospital charges among ICU admissions with and without sepsis. In order to facilitate comparisons to prior studies, we also examined hospital mortality among ICU admissions with sepsis, as well as among the subgroup with septic shock. In addition, we examined the contribution of sepsis to hospital length of stay, hospital charges, and short-term mortality among all ICU admissions with SLE.

### Study variables

Study variables were selected based on clinical plausibility and prior reports [12, 30, 36]. We abstracted data on patients’ age, gender, race/ethnicity, health insurance, comorbid conditions (based on the Deyo modification of the Charlson comorbidity index [37, 38]), transfer from another facility, admission during weekend, sites of infection (see Additional file 1), type and number of organ dysfunctions, All Patients Refined Diagnosis Related Groups (APR-DRG) severity of illness indicators, use of invasive mechanical ventilation (termed hereafter mechanical ventilation), hemodialysis (see Additional file 1), hospitals’ teaching status, hospital length of stay, hospital charges, hospital disposition, and year of hospitalization. Total hospital charges were adjusted for inflation using the consumer price index and reported as 2014 US dollars [39]. The TIPUDF and the state of Texas do not provide tools for conversion hospital charges to costs.

### Data analysis

We summarized categorical variables as numbers and percentages, while continuous variables were reported as mean (standard deviation [SD]) or median (interquartile [IQR]). The chi-squared test was used for group comparison involving categorical variables, while the Mann-Whitney test was used for comparison of continuous variables.

Because the TIPUDF dataset provides discharge-level, rather than patient-level information, precluding accounting for repeated admissions, we report the number of hospitalizations and ICU admissions as units of analysis, rather than the number of patients. The state of Texas masks gender data of hospitalizations with a diagnosis of infection with the human immunodeficiency virus, and of those with ethanol or drug abuse. Thus, analyses of gender were restricted to ICU admissions where gender data were not masked.

We used logistic regression to examine the temporal trends of ICU utilization, hospital mortality, and discharge to hospice and weighted least-squares regression to evaluate the temporal trends of hospital length of stay and hospital charges, using the calendar year as a predictor for each model.

In order to provide further anchoring context to the temporal trajectories of ICU utilization, hospital resource utilization and short-term mortality, we used weighted least-squares regression to examine the corresponding temporal trends of the burden of chronic illness and illness severity, using the Deyo comorbidity index and the number of organ dysfunctions, respectively, as proxy measures.

A multivariable linear regression model was fitted to quantify the independent contribution of sepsis to hospital length of stay among ICU admissions. A similar model was constructed to examine the association of sepsis with hospital charges among ICU admissions. The variables entered into the multivariable models included age, gender, race/ethnicity, health insurance, Deyo comorbidity index, transfer from another hospital, admission on weekend, teaching status of the hospital, number of organ dysfunctions, and sepsis. Model findings are reported as coefficients and standard errors (SE).

We used multivariable logistic regression modeling to examine the association of sepsis as independent predictor with short-term mortality as a dependent variable among ICU admissions, following examination for multicollinearity. Univariate logistic regressions were first carried out, with covariates with *p* < 0.1 considered for multivariate analysis. The multivariable logistic model included the following covariates: age, gender, race/ethnicity, health insurance, Deyo comorbidity index (adjusted after excluding the point score for connective tissue disease), transfer from another hospital, teaching status of the hospital, number of organ dysfunctions, sepsis, and year of admission. Covariates were entered using backward stepwise selection. We reported model findings as adjusted odds ratios (aOR) and 95% confidence intervals (95% CI) and calculated the adjusted difference in probability of short-term mortality between ICU admissions with and without sepsis, using empirical Bayesian posterior estimates from the multivariable logistic regression model. A similar multivariable model was constructed to identify independent predictors of short-term mortality among ICU admissions with sepsis. The covariates entered into the final model included age, gender, race/ethnicity, health insurance, Deyo comorbidity index, transfer from another hospital, number of organ dysfunctions, and type of infection. An alternative model was fitted with the covariates noted above, but using individual comorbidities and the type of organ dysfunctions instead of the Deyo comorbidity index and the number of organ dysfunctions, respectively, as predictors of short-term mortality among SLE ICU admissions with sepsis. The comparative accuracy of these two models (C-statistic) was examined using the method described by Hanley and McNeil [40].

To evaluate the robustness of the study findings on the predictors of short-term mortality among ICU admissions with sepsis, two sensitivity analyses were performed repeating separate multivariable regression modeling, employing the approach described above, using (a) only “explicit” sepsis codes and (b) defining sepsis using the Angus implementation [30].

Data management was performed using Excel and Access (Microsoft, Redmond, Washington) and statistical analyses were performed with MedCalc version 18 (MedCalc Software, Ostend, Belgium). A two-sided *p* value < 0.05 was considered statistically significant.

## Results

Among 94,338 hospitalizations with SLE, 34,991 (37.1%) were admitted to the ICU. Sepsis was reported in 17,037 (18.1%) of SLE hospitalizations and 9409 (55.2%) of those with sepsis had an ICU admission, with a diagnosis of sepsis present in 26.9% of all SLE admissions to the ICU. The annual volumes of all SLE hospitalizations, those with and without sepsis and of ICU admissions with and without sepsis, are detailed in Additional file 2.

### Cohort characteristics

The characteristics of ICU admissions with and without sepsis are outlined in Table 1. ICU admissions with sepsis were more commonly aged 65 years or older (25.3% vs. 21.4%, respectively), and less commonly white (35.6% vs. 39.1%, respectively) or privately insured (31.4% vs. 34.6%, respectively). Septic ICU admissions had a higher Deyo comorbidity index (3.1 vs. 2.4, respectively) and had more often three or more organ dysfunctions (24% vs. 4.7%, respectively). The most common sites of infection reported among septic ICU admissions were respiratory (46.6%), urinary (40.1%), and abdominal (13.6%). The mean hospital length of stay was markedly longer among ICU admissions with sepsis than among those without sepsis (11.6 days vs. 5.6 days, respectively), with nearly twofold higher mean hospital charges among the former ($126,037 vs. $61,176, respectively). Crude short-term mortality was markedly higher among ICU admissions with sepsis, as compared with those without sepsis (12.8% vs. 2.7%, respectively). Hospital mortality among ICU admissions with and without sepsis was 9.9% and 1.8%, respectively. Septic shock was reported in 1665 ICU admissions (17.7% of those with sepsis). The short-term mortality among ICU admissions with septic shock was 32.6%.

**Table 1:** The characteristics of ICU admissions with and without sepsis

Variables	Sepsis^a^	Non-sepsis^a^	*p*
	*n* = 9409	*n* = 25,582	
Age, years			< 0.0001
18–44	3338 (35.5)	9676 (37.8)	
45–64	3694 (39.3)	10,496 (41.0)	
≥ 65	2377 (25.3)	5410 (21.1)	
Gender^b^			
Female	7849 (88.6)	21,371 (89.4)	0.0379
Race/ethnicity			< 0.0001
White	3345 (35.6)	10,004 (39.1)	
Hispanic	2564 (27.5)	6362 (24.9)	
Black	2809 (29.9)	7595 (29.7)	
Other	691 (7.3)	1621 (6.3)	
Health insurance			< 0.0001
Private	2955 (31.4)	8841 (34.6)	
Medicare	4521 (48.0)	10,739 (42.0)	
Medicaid	1109 (11.8)	3161 (12.4)	
Uninsured	736 (7.8)	2531 (9.9)	
Other	84 (0.9)	294 (1.1)	
Missing	4 (0.04)	16 (0.06)	
Deyo comorbidity index^c^	3.1 (1.9)	2.4 (1.7)	< 0.0001
Selected comorbidities			
Chronic lung disease	2775 (29.5)	7033 (27.5)	0.0002
Congestive heart failure	3029 (28.9)	5448 (21.3)	< 0.0001
Cerebrovascular disease	821 (8.7)	2386 (9.3)	0.084
Renal disease	4865 (51.7)	7556 (29.5)	< 0.0001
Diabetes	2390 (25.4)	5907 (23.1)	< 0.0001
Malignancy	385 (4.1)	942 (3.7)	0.0754
Liver disease	981 (10.4)	1505 (5.9)	< 0.0001
Transfer from another hospital	841 (8.9)	1261 (4.9)	< 0.0001
Weekend admission	2199 (23.4)	5572 (21.8)	0.0015
Teaching hospital	2863 (30.4)	7269 (28.4)	0.0002
Site of infection		NA	
Respiratory	4381 (46.6)		
Urinary	3771 (40.1)		
Abdominal	1275 (13.6)		
Skin and soft tissue	664 (7.1)		
Device-related	561 (6.0)		
Other^d^	860 (9.1)		
Unknown	572 (6.1)		
APR-DRG SOI			< 0.0001
Minor	2 (< 0.1)	504 (2)	
Moderate	267 (2.8)	8901 (34.8)	
Major	4108 (43.7)	13,536 (52.9)	
Extreme	5032 (53.5)	2641 (10.3)	
Number of organ dysfunctions^c^	1.9 (1.1)	0.7 (0.9)	< 0.0001
Type of organ dysfunctions			
Respiratory	3692 (39.2)	2267 (8.9)	< 0.0001
Cardiovascular	1347 (14.3)	1750 (6.8)	< 0.0001
Renal	6748 (71.7)	8722 (34.1)	< 0.0001
Hepatic	438 (4.7)	267 (1.0)	< 0.0001
Hematological	2136 (22.7)	2176 (8.5)	< 0.0001
Neurological	1410 (15.0)	951 (3.7)	< 0.0001
Mechanical ventilation	2306 (24.5)	1139 (4.5)	< 0.0001
Hemodialysis	2449 (26.0)	3657 (14.3)	< 0.0001
Hospital length of stay			
Mean (SD)	11.6 (11.9)	5.6 (6.0)	
Median (IQR)	8 (5–14)	4 (2–7)	< 0.0001
Hospital charges			
Mean (SD)^c^	126,037 (154,503)	61,176 (74,303)	
Median (IQR)	77,389 (42,145–148,895)	39,834 (23,552–71,865)	< 0.0001
Hospital disposition			
Death	932 (9.9)	464 (1.8)	< 0.0001
Hospice	275 (2.9)	226 (0.9)	< 0.0001
Home	4403 (46.8)	18,726 (73.2)	< 0.0001
Home with home health services	1123 (11.9)	2416 (9.4)	< 0.0001
Another hospital	1436 (15.3)	1885 (7.4)	< 0.0001
Nursing facility	1092 (11.6)	1355 (5.3)	< 0.0001
Leave against medical advise	106 (1.2)	410 (1.6)	0.0008

*APR-DRG* All Patients Refined Diagnosis-Related Groups, *SOI* severity of illness *IQR* interquartile range

^a^Figures represent numbers and percentages unless specified otherwise. Percentage figures in parentheses may not add to 100 due to rounding

^b^Gender was reported for 8863 ICU admissions with sepsis and for 23,915 ICU admissions without sepsis; the percent figures for gender in each column refer to that column’s denominator for gender

^c^Mean (standard deviation [SD])

^d^Other sites of infection include endocarditis, genital, central nervous system, and bone

### Temporal trends of ICU admissions, characteristics, and outcomes

**Fig. 1:**
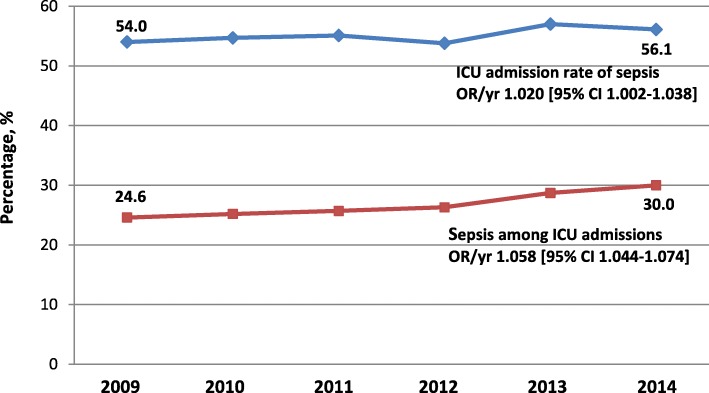
The annual rates of ICU admission among septic hospitalizations with systemic lupus erythematosus (SLE) and the fractions of sepsis among all ICU admission with SLE during 2009–2014. OR, odds ratio; yr, year; 95% CI, 95% confidence interval

The annual rate of ICU admissions among SLE hospitalizations sepsis rose from 54.0% to 56.1% between 2009 and 2014 (OR 1.020/year [95% CI 1.002–1.038]; *p* = 0.0293), while the fraction of ICU admissions with reported sepsis rose over the study period from 24.6% to 30% between 2009 and 2014, respectively (OR 1.058/year [95% CI 1.044–1.074]; *p* < 0.0001) (Fig. 1). The volume of ICU admissions among all SLE hospitalizations and among those with sepsis rose between 2009 and 2014 by 25.2% and 52.7%, respectively, and sepsis accounted for 51.5% of the growth in volume of ICU admissions during that period.

The annual increase in the Deyo comorbidity index and the number of organ dysfunctions among ICU admissions with sepsis was modest, though statistically significant (coefficient [95% CI], Deyo comorbidity index + 0.03 [0.01–0.05]; *p* = 0.0191; number of organ dysfunctions + 0.03 [0.02–0.05]; *p* < 0.0001).

Hospital length of stay decreased over time (− 0.21 days/year [95% CI from − 0.35 to − 0.06]; *p* = 0.0044), while hospital charges rose annually ($2184/year [95% CI 343–4024]; *p* = 0.0201) among SLE ICU admissions with sepsis.

Hospital mortality remained unchanged over time among septic ICU admissions (OR 1.012/year [95% CI 0.976–1.048]; *p* = 0.5111), while their rate of discharge to hospice increased from 1.5% to 3.5% between 2009 and 2014 (OR 1.130/year [95% CI 1.051–1.216]; *p* = 0.0010).

### The impact of sepsis on hospital resource utilization and short-term mortality among ICU admissions

The full model data on the association of sepsis with hospital length of stay, hospital charges, and short-term mortality among ICU admissions are detailed in Tables 2, 3, and 4, respectively. On adjusted analyses, sepsis was associated with hospital length of stay of additional 4.4 days and with additional hospital charges of $39,299.

**Table 2:** Univariate and multivariate regression analysis of the association of sepsis with hospital length of stay among ICU admissions

Variables	Unadjusted coefficient (SE)	*p*	Adjusted coefficient (SE)^a^	*p*
Age (years)				
18–44	Reference		Reference	
45–64	− 0.2196 (0.1027)	0.0326	− 0.2093 (0.1008)	0.0378
≥ 65	− 0.2070 (0.1213)	0.0878	− 0.3543 (0.1240)	0.0043
Gender				
Male	Reference		Reference	
Female	− 0.5979 (0.1509)	0.0001	− 0.2490 (0.1380)	0.0713
Race/ethnicity				
White	Reference		Reference	
Hispanic	+ 0.3199 (0.1122)	0.0044	+ 0.0339 (0.1100)	0.7576
Black	+ 0.5898 (0.1070)	< 0.0001	+ 0.3546 (0.1066)	0.0009
Other	+ 0.2157 (0.1273)	0.0318	+ 0.1728 (0.1035)	0.0921
Health insurance				
Private	Reference		Reference	
Medicare	− 0.1572 (0.1037)	0.1296	NA	
Medicaid	+ 0.5840 (0.1511)	0.0001	+ 0.8254 (0.1379)	< 0.0001
No insurance	+ 0.1102 (0.1673)	0.5100	NA	
Other	− 0.4364 (0.4422)	0.3236	NA	
Deyo comorbidity index				
1	Reference		Reference	
2–3	+ 1.1905 (0.1052)	< 0.0001	+ 0.0752 (0.1060)	0.4778
≥ 3	+ 2.6888 (0.1194)	< 0.0001	+ 0.6456 (0.1250)	< 0.0001
Transfer from another hospital	+ 5.9152 (0.1878)	< 0.0001	+ 4.3064 (0.1814)	< 0.0001
Weekend admission	− 0.3642 (0.1089)	0.0008	− 0.2984 (0.1040)	0.0041
Teaching hospital	+ 1.5480 (0.0994)	< 0.0001	+ 1.0423 (0.0962)	< 0.0001
Number of organ dysfunctions				
1	Reference		Reference	
2–3	+ 4.5096 (0.1277)	< 0.0001	+ 2.1771 (0.1452)	< 0.0001
≥ 3	+ 8.8733 (0.1528)	< 0.0001	+ 5.6673 (0.1766)	< 0.0001
Sepsis	+ 5.9923 (0.0969)	< 0.0001	+ 4.3556 (0.1125)	< 0.0001
Year of admission	− 0.0177 (0.0267)	0.5062	NA	

*SE* standard error

**Table 3:** Univariate and multivariate regression analysis of the association of sepsis with hospital charges among ICU admissions

Variables	Unadjusted coefficient (SE)	*p*	Adjusted coefficient (SE)	*p*
Age (years)			NA	
18–44	Reference			
45–64	+ 1089 (1289)	0.3981		
≥ 65	− 653 (1521)	0.6678		
Gender				
Male	Reference		Reference	
Female	− 10,826 (1900)	< 0.0001	− 5258 (1747)	0.0026
Race/ethnicity				
White	Reference		Reference	
Hispanic	+ 2622 (1409)	0.0627	+ 987 (1370)	0.471
Black	+ 1112 (1343)	0.4078	NA	
Other	+ 1492 (1510)	0.7832	NA	
Health insurance				
Private	Reference		Reference	
Medicare	− 2018 (1301)	0.1208	NA	
Medicaid	− 3146 (1896)	0.097	− 1304 (1867)	0.485
No insurance	− 13,075 (2098)	< 0.0001	− 8782 (2060)	< 0.0001
Other	− 1763 (5547)	0.7506	NA	
Deyo comorbidity index				
1	Reference		Reference	
2–3	+ 14,394 (1321)	< 0.0001	+ 1399 (1351)	0.3003
≥ 3	+ 30,788 (1500)	< 0.0001	+ 5673 (1576)	0.0003
Transfer from another hospital	+ 61,751 (2367)	< 0.0001	+ 40,665 (2296)	< 0.0001
Weekend admission	− 5386 (1366)	0.0001	− 3392 (1317)	0.01
Teaching hospital	+ 10,699 (1251)	< 0.0001	+ 6004 (1221)	< 0.0001
Number of organ dysfunctions				
1	Reference		Reference	
2–3	+ 449,361 (1588)	< 0.0001	+ 29,479 (1834)	< 0.0001
≥ 3	+ 123,284 (1901)	< 0.0001	+ 96,468 (2234)	< 0.0001
Sepsis	+ 68,862 (1233)	< 0.0001	+ 39,299 (1422)	< 0.0001
Year of admission	+ 2939 (335)	< 0.0001	+ 1680 (320)	< 0.0001

*SE* standard error

**Table 4:** Univariate and multivariate logistic regression analysis of predictors of short-term mortality among all ICU admissions

Variables	Unadjusted odds ratio	*p*	Adjusted odds ratio	*p*
	(95% CI)		(95% CI)	
Age (years)				
18–44	Reference		Reference	
45–64	1.785 (1.608–1.982)	< 0.0001	1.513 (1.498–1.831)	< 0.0001
≥ 65	2.593 (2.446–2.574)	< 0.0001	2.308 (2.156–2.773)	< 0.0001
Gender				
Male	Reference			
Female	0.662 (0.579–0.757)	< 0.0001	0.779 (0.665–0.913)	0.0021
Race/ethnicity				
White	Reference		Reference	
Hispanic	0.810 (0.720–0.912)	0.0005	NA	
Black	0.722 (0.643–0.810)	< 0.0001	0.813 (0.713–0.926)	0.002
Other	1.014 (0.845–1.217)	0.8773	NA	
Health insurance				
Private	Reference		Reference	
Medicare	1.437 (1.315–1.608)	< 0.0001	1.153 (1.055–1.395)	0.031
Medicaid	0.862 (0.509–0.911)	< 0.0001	NA	
No insurance	1.714 (1.479–1.833)	< 0.0001	1.265 (1.017–1.573)	0.0344
Other	0.912 (0.628–0.961)	0.0091	NA	
Deyo comorbidity index	1.338 (1.312–1.364)	< 0.0001	1.183 (1.152–1.214)	< 0.0001
Transfer from another hospital	2.521 (2.187–2.904)	< 0.0001	1.512 (1.269–1.803)	< 0.0001
Weekend admission	1.005 (0.899–1.123)	0.9229	NA	
Teaching hospital	1.212 (1.098–1.338)	0.0001	NA	
Number of organ dysfunctions	2.710 (2.612–2.812)	< 0.0001	1.839 (1.750–1.933)	< 0.0001
Sepsis	5.308 (4.818–5.848)	< 0.0001	1.505 (1.65–0.71)	< 0.0001
Year of admission	1.035 (1.007–1.064)	0.0132	NA	

Sepsis was associated with higher odds of short-term mortality (aOR 1.505 [95% CI 1.335–1.698]) among ICU admissions with SLE. The adjusted probability of short-term mortality was 13% (95% CI 12.6–13.3) among ICU admissions with sepsis and 2.7% (95% CI 2.6–2.8) among ICU admissions without sepsis.

ICU admissions with sepsis accounted for 43.2% of all hospital days and for 42.5% of the aggregate hospital charges among all ICU admissions with SLE. Finally, sepsis was reported in 66.8% of hospital deaths and in 63.6% of the short-term mortality events among ICU admissions.

### Predictors of short-term mortality among ICU admissions with sepsis

The univariate and multivariate analyses of the predictors of short-term mortality among ICU admissions with sepsis are detailed in Table 5 and Additional file 3. Using the Deyo comorbidity index and the number of organ dysfunctions (Additional file 3), the model’s C-statistic was 0.802 (95% CI 0.794–0.811). Following refitting of the model with individual comorbid conditions and the type of organ dysfunction (Table 5), the C-statistic was 0.837 (95% CI 0.829–0.844). The test for equality of the two models showed *p* = 0.0001, indicating better accuracy of the second model, with its key findings outlined below.

**Table 5:** Univariate and multivariate logistic regression analysis of predictors of short-term mortality among ICU admissions with sepsis

Variables	Unadjusted odds ratio	*p*	Adjusted odds ratio	*p*
	(95% CI)		(95% CI)	
Age (years)				
18–44	Reference		Reference	
45–64	1.524 (1.470–1.601)	< 0.0001	1.470 (1.403–1.537)	< 0.0001
≥ 65	2.422 (2.237–2.635)	< 0.0001	2.209 (2.185–2.410)	< 0.0001
Gender				
Male	Reference			
Female	0.690 (0.578–0.824)	< 0.0001	0.829 (0.675–1.018)	0.0738
Race/ethnicity				
White	Reference		Reference	
Hispanic	0.748 (0.641–0.871)	0.0002	NA	
Black	0.672 (0.577–0.783)	< 0.0001	0.848 (0.718–1.003)	0.0548
Other	0.962 (0.763–1.213)	0.7475	NA	
Health insurance				
Private	Reference		Reference	
Medicare	1.251 (0.771–1.029)	0.0849	NA	
Medicaid	0.673 (0.543–0.836)	0.0003	NA	
No insurance	1.642 (1.586–1.815)	< 0.0001	1.406 (1.075–1.840)	0.0128
Other	1.004 (0.801–1.258)	0.9672	NA	
Comorbid conditions				
Chronic lung disease	1.032 (0.904–1.178)	0.6351	NA	
Congestive heart failure	1.344 (1.188–1.524)	< 0.0001	NA	
Cerebrovascular disease	1.966 (1.643–2.353)	< 0.0001	1.569 (1.265–1.946)	< 0.0001
Renal disease	0.664 (0.587–0.750)	0.0001	NA	
Diabetes	0.678 (0.496–0.927)	0.015	0.725 (0.505–1.042)	0.083
Malignancy	3.633 (2.880–4.584)	< 0.0001	3.604 (2.725–4.767)	< 0.0001
Liver disease	2.885 (2.460–3.384)	< 0.0001	1.501 (1.128–1.997)	0.0053
Transfer from another hospital	1.918 (1.604–2.294)	< 0.0001	1.500 (1.216–1.851)	0.0002
Weekend admission	1.004 (0.871–1.158)	0.9472	NA	
Teaching hospital	1.096 (0.962–1.248)	0.1649	NA	
Infection site				
Respiratory	Reference			
Urinary	0.575 (0.489–0.676)	< 0.0001	0.759 (0.433–0.952)	0.0041
Abdominal	0.837 (0.675–1.083)	0.1556	NA	
Skin and soft tissue	0.702 (0.428–1.150)	0.1603	NA	
Devise-related	1.602 (0.873–2.971)	0.573	NA	
Other	2.116 (0.905–3.764)	0.9593	NA	
Type of organ dysfunction				
Respiratory	7.240 (6.265–8.368)	< 0.0001	6.152 (5.243–7.219)	< 0.0001
Cardiovascular	1.502 (1.284–1.757)	< 0.0001	1.418 (1.172–1.716)	0.0003
Renal	1.180 (1.027–1.354)	0.0188	1.410 (1.196–1.663)	< 0.0001
Hepatic	4.958 (4.049–6.070)	< 0.0001	1.895 (1.322–2.715)	0.0005
Hematological	2.318 (2.039–2.636)	< 0.0001	1.839 (1.573–2.149)	< 0.0001
Neurological	2.954 (2.570–3.394)	< 0.0001	1.798 (1.520–2.127)	< 0.0001
Year of admission	1.012 (1.976–1.048)	0.5111	NA	

The odds of short-term mortality rose with age, being over 2-fold higher among ICU admissions with sepsis aged 65 years or older, as compared with those aged 18–44 years. Lack of health insurance was associated with higher odds of short-term mortality (aOR 1.406 [95% CI 1.075–1.840]). Infection of the urinary tract was associated with lower odds of short-term mortality, as compared to that of the respiratory system (aOR 0.759 [95% CI 0.433–0.952]). Although female gender, as well as black race and Hispanic ethnicity, were strongly associated with lower odds of short-term mortality among septic ICU admissions on univariate analyses, these associations were not statistically significant on multivariate analyses. The short-term mortality among ICU admissions with sepsis did not change over time on adjusted analyses.

### Sensitivity analyses

The results of sensitivity analyses are detailed in Additional files 4 and 5. The findings of sensitivity analyses were generally consistent with the primary analyses of the predictors of short-term mortality of ICU admissions with sepsis. However, the odds of short-term mortality were significantly lower among females when sepsis definition was restricted to the Angus implementation, while no gender association with short-term mortality was noted when using only “explicit” sepsis codes.

## Discussion

### Key findings

In this population-based study, most SLE hospitalizations with sepsis were managed in the ICU and sepsis accounted for over half of the growth in ICU utilization among SLE hospitalizations. Care of critically ill SLE hospitalizations with sepsis was increasingly costly, accounting for nearly half of the aggregate hospital charges among all ICU admissions with SLE. The short-term mortality among critically ill SLE hospitalizations with sepsis was relatively low but remained unchanged over time, and sepsis was present in two thirds of all hospital deaths among ICU admissions with SLE.

### Relationship to previous studies

The demand for critical care resources among adult SLE hospitalizations with sepsis has not been previously examined, to our knowledge. However, the high and rising ICU utilization among SLE hospitalizations with sepsis can be placed in perspective by examination of the patterns of ICU admission among all SLE hospitalizations in the present and prior studies and comparing our findings among septic hospitalizations with their counterparts in the general population. The rate of admission to the ICU was markedly higher among all SLE hospitalizations in our cohort than that described by other investigators (37.1% vs. 4.9% [41] to 20% [20], respectively), likely reflecting in part the markedly lower availability of ICU beds outside the US [21, 42, 43] and differences in local practice.

The rate of ICU admission among septic SLE hospitalizations is comparable to that in prior reports in the general population in the US (55.2% vs. 54.7% [32], respectively). However, these comparable figures underestimate the considerably higher burden of sepsis among critically ill patients with SLE. Sepsis rates were markedly higher among SLE hospitalizations than in the general population in the US (18.1% vs. up to 9.4% [44], respectively), and sepsis was increasingly more common among ICU admissions with SLE (rising from 24.6% to 30% vs. 20.1% [15], respectively). Finally, sepsis accounted for over 50% of the growth in volume of ICU admissions among SLE hospitalizations during the study period. The findings of rising burden of chronic comorbidity and severity of illness do not support relaxation of the threshold for ICU admission among septic hospitalizations as a significant source of their growing ICU utilization. Our findings extend prior reports on the markedly higher rates of infectious complications in SLE [7] and specifically among ICU admissions [6].

The present study provides, to our knowledge, the first population-level quantification of the economic toll of sepsis among critically ill patients with SLE. Septic ICU admissions incurred nearly extra $40,000 in hospital charges, as compared to those without sepsis. Moreover, care of septic ICU admissions became increasingly costly over time, though the associated hospital length of stay decreased. The finding of unchanged short-term mortality over time among septic ICU admissions does not support this factor as a contributor to the reduced hospital length of stay. On the other hand, the rising burden of chronic illness and illness severity over time likely contributed to the rising hospital charges. Thus, our findings of decreasing hospital length of stay, coupled with rising hospital charges, suggest increasing care intensity. Finally, sepsis was associated with nearly half of all hospital days and aggregate hospital charges among ICU admissions with SLE.

Hospital mortality among septic ICU admissions with SLE was the lowest reported to date (9.9% vs. 31% [21] to 64% [11], respectively). Our results likely reflect differences in baseline patient characteristics and care of SLE, more selective triage to the ICU due to lower availability of ICU beds outside the US [21, 42, 43], and possibly improved critical care over time. Nevertheless, despite their relatively low short-term mortality, septic ICU admissions had nearly fivefold higher adjusted probability of death, as compared to non-septic ICU admissions. The latter difference between septic vs. non-septic critically ill SLE hospitalizations is substantially higher than the twofold higher hospital mortality reported among ICU admissions with and without sepsis in the general population [45]. The factors underlying the markedly greater adverse prognostic impact of sepsis vs. those without sepsis among critically ill hospitalizations with SLE vs. the corresponding figures in the general population are unclear. However, these differences may reflect in part some of the greater challenges in early recognition and care of sepsis in SLE (see also further discussion below). Sepsis was reported in two out of three hospital deaths among ICU admissions. Finally, the short-term mortality among septic shock patients in the present study was similar to that reported in a national cohort in France during 2010–2015, with comparable case definition (32.6% vs. 30.9% [24], respectively).

Short-term mortality may be expected to be lower among SLE patients with sepsis than that in the general population due to the younger age and comparatively lower burden of age-related comorbidities among the former. On the other hand, it has been hypothesized that sepsis outcomes in SLE patients may be worse due to their prevalent immune dysfunction [23]. Short-term mortality among septic ICU admissions in our study was lower than among patients with sepsis in the general population in a recent US study using similar ICD code-based case definitions (12.8% vs. 16–18% [32], respectively). Similarly, hospital mortality was lower in the subgroup with septic shock in the present cohort, as compared with that in the general US population, using similar ICD-9 code (32.6% vs. 42.2% [46], respectively). However, only two studies have compared, to our knowledge, the outcomes of septic ICU patients with and without SLE within the same cohort. No difference in 30-day mortality was found in a recent registry-based study [21]. In another single-center study, the adjusted odds of 30-day mortality were 27% lower among septic patients with an autoimmune disease than among those without and 25% lower among the subgroup with SLE, though the finding was not statistically significant for the latter [23]. Further studies are needed to better characterize the host response to sepsis among SLE patients.

A novel key finding of the present study is the unchanged short-term mortality over time among septic ICU admissions with SLE. This finding contrasts the progressive decrease in mortality in the majority of studies on sepsis in the general population, in both administrative data and clinical studies [47]. These conflicting trends occurred despite the rise in illness severity over time in both the present study and in the general population [48]. However, data on sepsis-related and general processes of care and their appropriateness in the present cohort are not available in the claims-based data used in the present study. Thus, studies of more granular data are needed to examine whether and to what extent do sepsis-related care processes differ between septic patients with SLE and those in the general population. Such data can inform future preventive and interventional efforts to improve the outcomes of sepsis in the SLE population. In the interim, it may be hypothesized that potential contributors to our findings may include the persistence of considerable challenges in early distinction between sepsis and SLE flare-up, with resultant delays in time-sensitive care elements and, possibly, increased risk of infection with multidrug-resistant pathogens due to more frequent exposure of SLE patients to antibiotics.

Lack of health insurance was associated with higher odds of death, as compared with private insurance, among septic critically ill hospitalizations with SLE. This finding is consistent with prior reports of increased mortality among critically ill patients lacking health insurance in general [49] and among those with sepsis in the general population [50]. Lack of health insurance has likely adversely affected patients’ access to primary and specialty care and thus may have led to worse control of SLE and other comorbidities. In addition, lack of health insurance may have led to economically-driven delays in seeking timely care for acute illness. Such delays may result in more severe illness on presentation with sepsis. Thus, the development and implementation of health care policy geared to expand the availability of adequate health insurance may help improving health outcomes of sepsis in SLE.

Our finding of reduced odds of short-term mortality among septic SLE hospitalizations admitted to the ICU who had urinary tract infections (UTI), as compared with those having an infection of the respiratory tract, is consistent with prior studies of sepsis in the general population [51, 52]. The factors underlying the more favorable outcome among septic patients with UTI remain uncertain. However, it has been suggested that this observation may be related to the structure of the bladder and the genitourinary tract as well as the washout by micturition [51] which may prevent bacterial invasion and limit absorption of microbes and bacterial toxins, and possibly due to more rapid recognition of UTI and relative ease of source control in obstructive uropathy.

Our study findings add to the ongoing uncertainties about the impact of gender and race/ethnicity on patients’ mortality in sepsis. Although the odds of short-term mortality among septic ICU admissions were markedly lower among females and racial/ethnic minorities (black and Hispanic), these findings were no longer significant on adjusted analyses. These findings, as well as those on sensitivity analysis of the subgroup with septic shock, are consistent with the recent report on septic shock among SLE patients in France [24]. On the other hand, the odds of short-term mortality were significantly lower among females, when sepsis was defined by the Angus implementation, which captures less severely ill population [32].

Prior studies in the general population have reported conflicting findings, with either increased [53], decreased [54], or neutral [55] effect of female gender on risk of sepsis-associated death and, similarly, greater [56], lower [57], or neutral [58] impact of minority race/ethnicity. The research on the underlying factors affecting gender-related sepsis outcomes focused on biological gender differences [59], while that exploring the role of race and ethnicity expanded investigations beyond biological factors into the roles of socioeconomic status, health-related behaviors, access to healthcare, and process of care delivery [58, 59]. Some of the challenges to better understanding of the drivers of disparate health outcomes across different race and ethnicity groups start with the basic application of these terms in practice. An increasing number of patients in the US define themselves as mixed race, though only one race is entered into health records. Overall, race represents exposure to health risks driven by biological (genetic), social, behavioral, and environmental factors. Ethnicity, in turn, is an artificial construct, which is defined by shared language, culture, and/or national origin, and it overlaps with race [60]. Although prior studies repeatedly highlighted race/ethnicity-related healthcare disparities in sepsis [61, 62], the underlying mechanisms of these findings remain incompletely understood. Thus, patterns of human genome DNA across races do not appear to explain health disparities in sepsis [60]. On the other hand, socioeconomic factors, which have not been consistently explored in prior studies, may potentially explain the race- and ethnicity-related health disparities in sepsis, as they are related with other risk factors for sepsis, such as smoking, alcohol use, comorbidities, access to health care, and differential aspects of healthcare delivery across race/ethnicity groups and merit further study. The present uncertainties continue to affect the examination of gender- and race/ethnicity-focused preventive and therapeutic interventions targeting in sepsis.

### Strengths and limitations

The present study is the largest to date on adult SLE patients with sepsis in the ICU. The use of a statewide, all-payer, high-quality dataset of consecutive hospitalizations allowed transcending of local variations in case mix and practice patterns. In addition, the large number of hospitalizations permitted more comprehensive examination of the spatial and temporal aspects of the epidemiology and outcomes of septic ICU patients.

However, our study has several important limitations, in addition to those mentioned earlier, related predominantly to its retrospective design and use of administrative data. First, because patient groups were identified based on ICD codes, there is a potential for misclassification, despite the use of established search algorithms. More specifically, inconsistency in the use of sepsis ICD codes may have occurred, affecting the proper classification of sepsis vs. non-sepsis hospitalizations. Such misclassification would be expected to blur the differences between sepsis and non-sepsis groups. Thus, the findings of the already markedly higher Deyo comorbidity index, number of organ dysfunctions, and short-term mortality in the septic ICU admissions, as compared with those classified non-septic may have been an underestimate of the actual difference between the groups. However, it is unlikely that such coding variation differs systematically between patients with SLE and those in the general population. Thus, the finding of divergent temporal trends in short-term mortality between the critically ill septic hospitalizations with SLE in the present cohort and those in the general population, when using identical ICD-based algorithms, does not support a major impact of variation in code use and resultant misclassifications. Second, the administrative data do not include information on patients’ duration of illness, immunomodulating therapy, disease activity, the type of sepsis (e.g., community-acquired, healthcare-associated, or hospital-acquired), and processes of care and their timeliness, all of which may have affected our findings. Thus, although the predictive models in this study adjusted for numerous covariates, residual confounding cannot be excluded. Last, the generalizability of our findings to other states, nationally, and to other countries is unknown.

## Conclusions

There is substantial demand for critical care services among SLE hospitalizations with sepsis, with the latter being a major driver of the rising ICU utilization among SLE hospitalizations. Septic ICU admissions are increasingly costly, accounting for a major share of hospital days and aggregate charges among critically ill SLE hospitalizations. Although the short-term mortality was relatively low among septic ICU admissions, it was associated with two thirds of all hospital deaths among ICU admissions with SLE and remained unchanged over time. Further studies are needed to corroborate the present findings, further examine the prognostic impact of SLE-related patient- and therapy-related characteristics, and to explore strategies to improve outcomes of sepsis in the SLE population.

## Supplementary information

**Additional file 1.** ICD-9 codes for sites of infection, mechanical ventilation and hemodialysis.

**Additional file 2.** The annual volume of hospitalization and ICU admission among hospitalizations with and without sepsis.

**Additional file 3.** Univariate and multivariate logistic regression analysis of predictors of short-term mortality among ICU admissions with sepsis, using the Deyo comorbidity index and the number of organ dysfunctions.

**Additional file 4.** Univariate and multivariate logistic regression analysis of predictors of short-term mortality among ICU admissions with sepsis, identified by “explicit” sepsis codes, using the Deyo comorbidity index and the number of organ dysfunctions.

**Additional file 5.** Univariate and multivariate logistic regression analysis of predictors of short-term mortality among ICU admissions with sepsis, identified by the Angus implementation, using the Deyo comorbidity index and the number of organ dysfunctions.

## Data Availability

The dataset analyzed during the current study is available through the Texas Department of State Health Services, Center for Health Statistics, Austin, Texas, at http://www.dshs.state.tx.us/thcic/hospitals/Inpatientpudf.shtm.

## Abbreviations

ICD-9-CM: International Classification of Diseases, Ninth Revision, Clinical Modification
SLE: Systemic lupus erythematosus
TIPUDF: Texas Inpatient Public Use Data File US United States
