# Supplementary material for: Epidemiology and outcomes of sepsis among hospitalizations with systemic lupus erythematosus admitted to the ICU: a population-based cohort study
Source: J Intensive Care. 2020 Jan 6;8:3. doi: 10.1186/s40560-019-0424-y (PMC6945625; doi:10.1186/s40560-019-0424-y)
Supplement: Supplementary file 1 — Additional file 1. ICD-9 codes for sites of infection, mechanical ventilation and hemodialysis. [file 40560_2019_424_MOESM1_ESM.docx]

**eTable 1. International Classification of Diseases, Ninth Edition, Clinical Modification (ICD 9 -CM) codes for sites of infection, mechanical ventilation and hemodialysis (Where only 3 or 4-digit codes are listed, all associated subcodes are included)**

**Category ICD-9-CM codes**

**Sites of infection**

Respiratory 481-486, 510, 513

Blood 790.7, 572.1, 673.3

Endocarditis 112.81, 421

Central nervous system 320, 322, 324, 325

Gastrointestinal/abdominal 003, 008, 540-542, 530.4, 530.86, 562.01, 562.03, 562.11, 562.13, 566, 567, 569.5, 569.83, 572.0, 575.0, 531.1, 531.2, 531.5, 531.6 532.1, 532.2, 532.5, 532.6, 533.1, 533.2, 533.5, 533.6, 534.1, 534.2, 534.5, 534.6

Urinary 590, 599.0

Genital 615, 634.0, 635.0, 636.0, 637.0, 638.0, 639.0, 646.6 658.4

Skin and soft tissue 675.1, 680, 682, 686, 998.5

Bone and joint 711.0, 730

Device-related 996.6

**Procedures**

Mechanical ventilation 96.70-96.72

Hemodialysis 38.95, 39.95
