# Supplementary material for: Epidemiology and outcomes of sepsis among hospitalizations with systemic lupus erythematosus admitted to the ICU: a population-based cohort study
Source: J Intensive Care. 2020 Jan 6;8:3. doi: 10.1186/s40560-019-0424-y (PMC6945625; doi:10.1186/s40560-019-0424-y)
Supplement: Supplementary file 2 — Additional file 2. The annual volume of hospitalization and ICU admission among hospitalizations with and without sepsis. [file 40560_2019_424_MOESM2_ESM.docx]

| **eTable 2. The annual volume of hospitalization and ICU admission among hospitalizations with and without sepsis** | | | | | | | | | |
| --- | --- | --- | --- | --- | --- | --- | --- | --- | --- |
|  |  |  |  |  | |  |  |  |  |
|  |  |  |  | **Year** | |  |  |  |  |
| **Category** |  | **2009** | **2010** | **2011** | **2012** | **2013** | **2014** |  |  |
| **All hospitalizations** |  | 14,098 | 14,482 | 15,916 | 16,070 | 16,506 | 17,266 |  |  |
| Hospitalizations with sepsis | | 2,331 | 2,413 | 2,721 | 2,970 | 3,180 | 3,422 |  |  |
| Hospitalizations without sepsis | | 11,767 | 12,069 | 13,195 | 13,100 | 13,326 | 13,844 |  |  |
| **All ICU admissions** |  | 5,106 | 5,244 | 5,840 | 6,085 | 6,323 | 6,394 |  |  |
| ICU admissions with sepsis | | 1,258 | 1,319 | 1,498 | 1,599 | 1,814 | 1,921 |  |  |
| ICU admissions without sepsis | | 3,848 | 3,925 | 4,342 | 4,486 | 4,509 | 4,473 |  |  |
