# Supplementary material for: Epidemiology and outcomes of sepsis among hospitalizations with systemic lupus erythematosus admitted to the ICU: a population-based cohort study
Source: J Intensive Care. 2020 Jan 6;8:3. doi: 10.1186/s40560-019-0424-y (PMC6945625; doi:10.1186/s40560-019-0424-y)
Supplement: Supplementary file 3 — Additional file 3. Univariate and multivariate logistic regression analysis of predictors of short-term mortality among ICU admissions with sepsis, using the Deyo comorbidity index and the number of organ dysfunctions. [file 40560_2019_424_MOESM3_ESM.docx]

| \| \| **eTable 3. Univariate and multivariate logistic regression analysis of predictors of short-term mortality** \| \| \| --- \| --- \| \| **among ICU admissions with sepsis, using the Deyo comorbidity index and the number of organ** \| \| \| \| **dysfunctions** \| \| \| \| \|  \| \| --- \| --- \| --- \| --- \| --- \| --- \| --- \| --- \| --- \| --- \| --- \| \|  \| \| \| \| \| \|  \| \|  \|  \|  \| \|  \|  \|  \|  \|  \| \|  \| **Unadjusted odds ratio** \|  \| **Adjusted odds ratio** \|  \| \| **Variables** \| **(95% CI)** \| **p** \| **(95% CI)** \| **p** \| \| **Age (years)** \|  \|  \|  \|  \| \| 18-44 \| Reference \|  \| Reference \|  \| \| 45-64 \| 1.524 (1.470-1.601) \| <0.0001 \| 1.488 (1.401-1.590) \| <0.0001 \| \| ≥65 \| 2.422 (2.237-2.635) \| <0.0001 \| 2.173 (1.952-2.318) \| <0.0001 \| \| **Gender** \|  \|  \|  \|  \| \| Male \| Reference \|  \|  \|  \| \| Female \| 0.690 (0.578-0.824) \| <0.0001 \| 0.864 (0.694-0.031) \| 0.0987 \| \| **Race/ethnicity** \|  \|  \|  \|  \| \| White \| Reference \|  \| Reference \|  \| \| Hispanic \| 0.748 (0.641-0.871) \| 0.0002 \| NA \|  \| \| Black \| 0.672 (0.577-0.783) \| <0.0001 \| 0.892 (0.629- 1.047) \| 0.0803 \| \| Other \| 0.962 (0.763-1.213) \| 0.7475 \| NA \|  \| \| **Health insurance** \|  \|  \|  \|  \| \| Private \| Reference \|  \| Reference \|  \| \| Medicare \| 1.251 (0.771-1.029) \| 0.0849 \| NA \|  \| \| Medicaid \| 0.673 (0.543-0.836) \| 0.0003 \| NA \|  \| \| No insurance \| 1.642 (1.586-1.815) \| <0.0001 \| 1.520 (1.170-1.974) \| 0.0017 \| \| Other \| 1.004 (0.801-1.258) \| 0.9672 \| NA \|  \| \| **Deyo comorbidity index^a^** \| 1.207 (1.174-1.241) \| <0.0001 \| 1.091 (1.054-1.129) \| <0.0001 \| \| **Transfer from another hospital** \| 1.918 (1.604-2.294) \| <0.0001 \| 1.714 (1.398-2.103) \| <0.0001 \| \| **Weekend admission** \| 1.004 (0.871-1.158) \| 0.9472 \| NA \|  \| \| **Teaching hospital** \| 1.096 (0.962-1.248) \| 0.1649 \| NA \|  \| \| **Infection site** \|  \|  \|  \|  \| \| Respiratory \| Reference \|  \|  \|  \| \| Urinary \| 0.575 (0.489-0.676) \| <0.0001 \| 0.643 (0.540-0.767) \| <0.0001 \| \| Abdominal \| 0.837 (0.675-1.083) \| 0.1556 \| NA \|  \| \| Skin and soft tissue \| 0.702 (0.428-1.150) \| 0.1603 \| NA \|  \| \| Devise-related \| 1.602 (0.873-2.971) \| 0.573 \| NA \|  \| \| Other \| 2.116 (0.905-3.764) \| 0.9593 \| NA \|  \| \| **Number of organ dysfunctions^b^** \| 2.177 (2.068-2.291) \| <0.0001 \| 2.082 (1.969-2.201) \| <0.0001 \| \| **Year of admission** \| 1.012 (1.976-1.048) \| 0.5111 \| NA \|  \| \| a odds ratio for increase by 1 point of the Deyo comorbidity index \| \| \|  \|  \| \| b odds ratio for increase by 1 additional organ dysfunction \| \|  \|  \|  \| |
| --- | --- | --- | --- | --- | --- | --- | --- | --- | --- | --- | --- | --- | --- | --- | --- | --- | --- | --- | --- | --- | --- | --- | --- | --- | --- | --- | --- | --- | --- | --- | --- | --- | --- | --- | --- | --- | --- | --- | --- | --- | --- | --- | --- | --- | --- | --- | --- | --- | --- | --- | --- | --- | --- | --- | --- | --- | --- | --- | --- | --- | --- | --- | --- | --- | --- | --- | --- | --- | --- | --- | --- | --- | --- | --- | --- | --- | --- | --- | --- | --- | --- | --- | --- | --- | --- | --- | --- | --- | --- | --- | --- | --- | --- | --- | --- | --- | --- | --- | --- | --- | --- | --- | --- | --- | --- | --- | --- | --- | --- | --- | --- | --- | --- | --- | --- | --- | --- | --- | --- | --- | --- | --- | --- | --- | --- | --- | --- | --- | --- | --- | --- | --- | --- | --- | --- | --- | --- | --- | --- | --- | --- | --- | --- | --- | --- | --- | --- | --- | --- | --- | --- | --- | --- | --- | --- | --- | --- | --- | --- | --- | --- | --- | --- | --- | --- | --- | --- | --- | --- | --- | --- | --- | --- | --- | --- | --- | --- | --- | --- | --- | --- | --- | --- | --- | --- | --- | --- | --- | --- | --- | --- | --- | --- | --- | --- | --- | --- | --- | --- | --- | --- |
|  |
