# Supplementary material for: Epidemiology and outcomes of sepsis among hospitalizations with systemic lupus erythematosus admitted to the ICU: a population-based cohort study
Source: J Intensive Care. 2020 Jan 6;8:3. doi: 10.1186/s40560-019-0424-y (PMC6945625; doi:10.1186/s40560-019-0424-y)
Supplement: Supplementary file 4 — Additional file 4. Univariate and multivariate logistic regression analysis of predictors of short-term mortality among ICU admissions with sepsis, identified by “explicit” sepsis codes, using the Deyo comorbidity index and the number of organ dysfunctions. [file 40560_2019_424_MOESM4_ESM.docx]

| **eTable 4. Univariate and multivariate logistic regression analysis of predictors of** | | | |  |
| --- | --- | --- | --- | --- |
| **short-term mortality among ICU admissions with sepsis, identified by "explicit" sepsis** | | | |  |
| **codes, and using the types of comorbidities and organ dysfunctions** | | |  |  |
|  |  |  |  |  |
|  | **Unadjusted odds ratio** |  | **Adjusted odds ratio** |  |
| **Variables** | **(95% CI)** | **p** | **(95% CI)** | **p** |
| **Age (years)** |  |  |  |  |
| 18-44 | Reference |  | Reference |  |
| 45-64 | 1.705 (1.587-1.821) | <0.0001 | 1.624 (1.589-1.692) | <0.0001 |
| ≥65 | 2.895 (2.802-2.978) | <0.0001 | 2.688 (2.608-2.725) | <0.0001 |
| **Gender** |  |  |  |  |
| Male | Reference |  |  |  |
| Female | 0.675 (0.522-0.873) | 0.0028 | NA |  |
| **Race/ethnicity** |  |  |  |  |
| White | Reference |  | Reference |  |
| Hispanic | 0.875 (0.699-1.094) | 0.2418 | NA |  |
| Black | 0.947 (0.761-1.178) | 0.6278 | NA |  |
| Other | 1.067 (.765-1.490) | 0.6993 | NA |  |
| **Health insurance** |  |  |  |  |
| Private | Reference |  | Reference |  |
| Medicare | 1.475 (0.790-1.084) | 0.1072 | NA |  |
| Medicaid | 0.6607 (0.494-0.882) | 0.0051 | NA |  |
| No insurance | 1.756 (1.690-1.910) | 0.0005 | 1.633 (1.109-2.405) | 0.013 |
| Other | 0.938 (0.407-2.126) | 0.8703 | NA |  |
| **Comorbid conditions** |  |  |  |  |
| Chronic lung disease | 0.836 (0.682-1.025) | 0.0863 | 0.698 (0.548-0.891) | 0.0038 |
| Congestive heart failure | 1.516 (1.258-1.828) | <0.0001 | NA |  |
| Cerebrovascular disease | 2.047 (1.529-2.740) | <0.0001 | 1.394 (0.991-1.959) | 0.0558 |
| Renal disease | 1.085 (0.9293-1.322) | 0.2521 | NA |  |
| Diabetes | 0.7938 (0.487-1.293) | 0.353 | NA |  |
| Malignancy | 2.779 (1.851-4.173) | <0.0001 | 2.723 (1.692-4.381) | <0.0001 |
| Liver disease | 2.887 (2.293-3.635) | <0.0001 | 2.128 (1.606-2.820) | <0.0001 |
| **Transfer from another hospital** | 1.933 (1.473-2.536) | <0.0001 | 1.760 (1.279-2.421) | 0.0005 |
| **Weekend admission** | 1.010 (0.821-1.241) | 0.925 | NA |  |
| **Teaching hospital** | 1.205 (1.002-1.449) | 0.0469 | NA |  |
| **Infection site** |  |  |  |  |
| Respiratory | Reference |  |  |  |
| Urinary | 0.662 (0.511-0.857) | 0.0018 | 0.817 (0.593-0.937) | 0.0059 |
| Abdominal | 1.153 (0.852-1.560) | 0.356 | NA |  |
| Skin and soft tissue | 0.4129 (0.239-0.712) | 0.0015 | NA |  |
| Devise-related | 0.563 (0.270-1.174) | 0.1259 | NA |  |
| Other | 1.450 (1.160-1.811) | 0.0011 | NA |  |
| **Type of organ dysfunction** |  |  |  |  |
| Respiratory | 6.776 (5.381-8.533) | <0.0001 | 6.617 (5.141-8.518) | <0.0001 |
| Cardiovascular | 1.529 (1.194-1.959) | 0.0008 | 1.313 (0.975-1.769) | 0.0721 |
| Renal | 1.860 (1.482-2.335) | <0.0001 | 1.535 (1.176-2.003) | 0.0016 |
| Hepatic | 3.990 (3.024-5.263) | <0.0001 | NA |  |
| Hematological | 2.371 (1.972-2.851) | <0.0001 | 1.687 (1.355-2.101) | <0.0001 |
| Neurological | 2.018 (1.653-2.463) | <0.0001 | NA |  |
| **Year of admission** | 0.950 (0.902-1.001) | 0.0529 | 0.938 (0.889-1.025) | 0.0549 |
