# Supplementary material for: Epidemiology and outcomes of sepsis among hospitalizations with systemic lupus erythematosus admitted to the ICU: a population-based cohort study
Source: J Intensive Care. 2020 Jan 6;8:3. doi: 10.1186/s40560-019-0424-y (PMC6945625; doi:10.1186/s40560-019-0424-y)
Supplement: Supplementary file 5 — Additional file 5. Univariate and multivariate logistic regression analysis of predictors of short-term mortality among ICU admissions with sepsis, identified by the Angus implementation, using the Deyo comorbidity index and the number of organ dysfunctions. [file 40560_2019_424_MOESM5_ESM.docx]

| **eTable 5. Univariate and multivariate logistic regression analysis of predictors of** | | | |  |
| --- | --- | --- | --- | --- |
| **short-term mortality among ICU admissions with sepsis, identified by the Angus implementation** | | | | |
| **and using the types of comorbidities and organ dysfunctions** | |  |  |  |
|  |  |  |  |  |
|  | **Unadjusted odds ratio** |  | **Adjusted odds ratio** |  |
| **Variables** | **(95% CI)** | **p** | **(95% CI)** | **p** |
| **Age (years)** |  |  |  |  |
| 18-44 | Reference |  | Reference |  |
| 45-64 | 1.413 (1.351-1.406) | <0.0001 | 1.305 (1.251-1.387) | <0.0001 |
| ≥65 | 1.892 (1.741-1.977) | <0.0001 | 1.824 (1.728-1.927) | <0.0001 |
| **Gender** |  |  |  |  |
| Male | Reference |  |  |  |
| Female | 0.702 (0.579-0.852) | 0.0003 | 0.790 (0.635-0.983) | 0.0349 |
| **Race/ethnicity** |  |  |  |  |
| White | Reference |  | Reference |  |
| Hispanic | 0.764 (0.648-0.901) | 0.0014 | NA |  |
| Black | 0.610 (0.515-0.722) | <0.0001 | 0.839 (0.701-1.004) | 0.0554 |
| Other | 0.924 (0.716-1.193) | 0.5474 | NA |  |
| **Health insurance** |  |  |  |  |
| Private | Reference |  | Reference |  |
| Medicare | 1.154 (0.829-1.075) | 0.0935 | NA |  |
| Medicaid | 0.697 (0.553-0.880) | 0.0024 | NA |  |
| No insurance | 1.461 (1.408-1.639) | <0.0001 | 1.291 (1.059-1.727) | 0.0183 |
| Other | 1.038 (0.894-1.419) | 0.6352 | NA |  |
| **Comorbid conditions** |  |  |  |  |
| Chronic lung disease | 1.094 (0.949-1.260) | 0.2132 | NA |  |
| Congestive heart failure | 1.354 (1.183-1.551) | <0.0001 | NA |  |
| Cerebrovascular disease | 1.903 (1.567-2.311) | <0.0001 | 1.532 (1.221-1.921) | 0.0002 |
| Renal disease | 0.638 (0.558-0.728) | <0.0001 | NA |  |
| Diabetes | 0.656 (0.466-0.924) | 0.0161 | 0.693 (0.466-1.031) | 0.0706 |
| Malignancy | 3.766 (2.947-4.182) | <0.0001 | 3.472 (2.603-4.632) | <0.0001 |
| Liver disease | 2.711 (2.274-3.233) | <0.0001 | 1.475 (1.092-1.991) | 0.0111 |
| **Transfer from another hospital** | 2.046 (1.692-2.475) | <0.0001 | 1.563 (1.253-1.949) | 0.0001 |
| **Weekend admission** | 1.056 (0.906-1.232) | 0.4818 | NA |  |
| **Teaching hospital** | 1.033 (0.895-1.191) | 0.6549 | NA |  |
| **Infection site** |  |  |  |  |
| Respiratory | Reference |  |  |  |
| Urinary | 0.574 (0.488-0.677) | <0.0001 | 0.826 (0.502-0.986) | 0.007 |
| Abdominal | 0.816 (0.656-1.014) | 0.0666 | NA |  |
| Skin and soft tissue | 0.849 (0.604-1.171) | 0.2054 | NA |  |
| Devise-related | 1.793 (0.709-2.403) | 0.7815 | NA |  |
| Other | 2.495 (0.866-4.038) | 0.9207 | NA |  |
| **Type of organ dysfunction** |  |  |  |  |
| Respiratory | 6.831 (5.846-7.981) | <0.0001 | 5.993 (5.059-7.100) | <0.0001 |
| Cardiovascular | 1.420 (1.196-1.686) | 0.0001 | 1.415 (1.156-1.732) | 0.0008 |
| Renal | 1.088 (0.938-1.263) | 0.2621 | NA |  |
| Hepatic | 4.744 (3.792-5.934) | <0.0001 | 2.167 (1.484-3.165) | 0.0001 |
| Hematological | 2.248 (1.955-2.585) | <0.0001 | 1.961 (1.663-2.312) | <0.0001 |
| Neurological | 2.906 (2.499-3.379) | <0.0001 | 1.935 (1.621-2.309) | <0.0001 |
| **Year of admission** | 1.025 (0.986-1.066) | 0.1985 | NA |  |
